# Supplementary figures and images for: Exogene: A performant workflow for detecting viral integrations from paired-end next-generation sequencing data
Source: PLoS One. 2021 Sep 22;16(9):e0250915. doi: 10.1371/journal.pone.0250915 (PMC8457494; doi:10.1371/journal.pone.0250915)

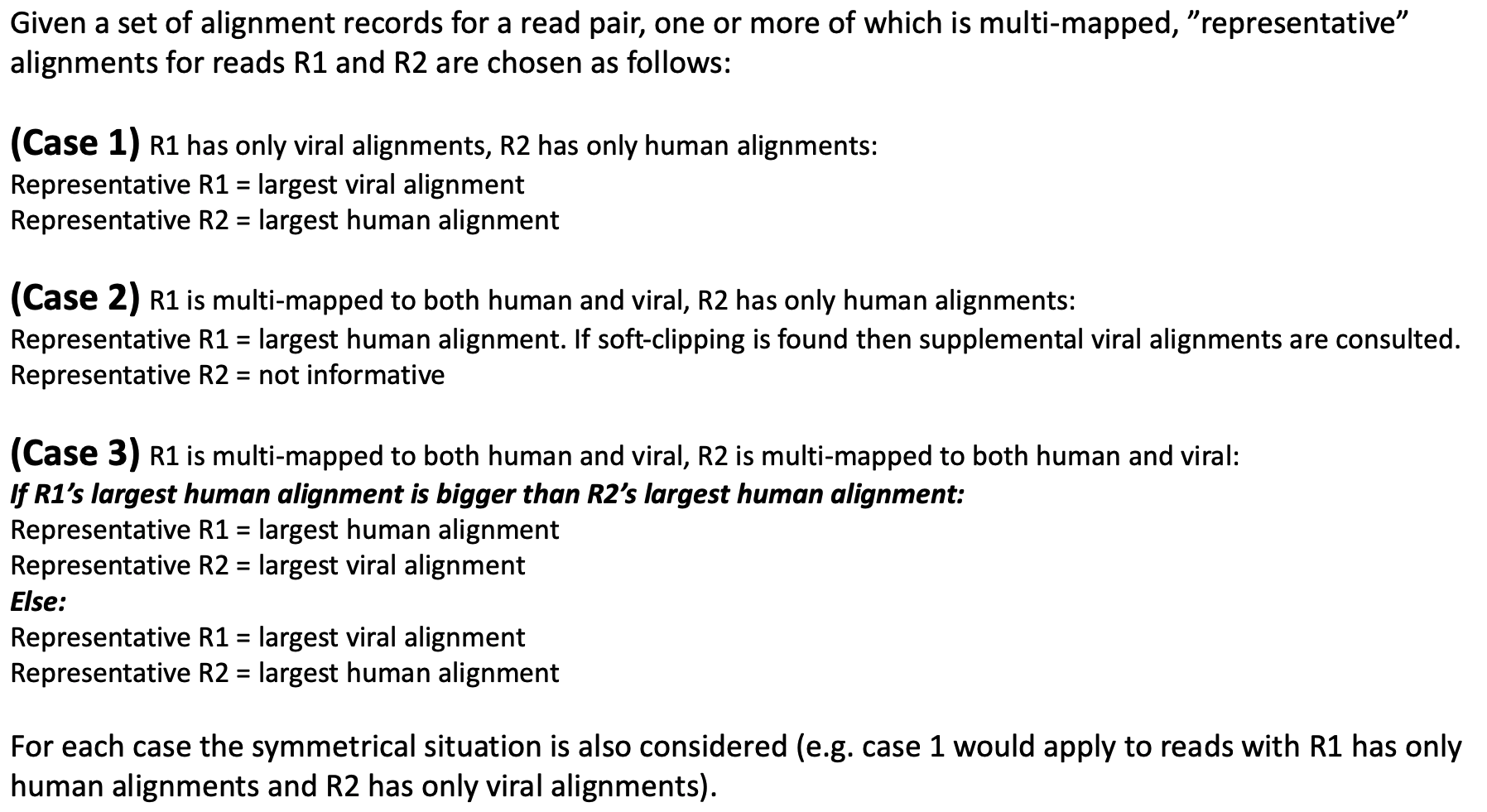

Supplement: S1 Fig — (TIFF) [file pone.0250915.s001.tiff]
